# On Predicting Optimal Structural Topologies in the Presence of Random Loads

**DOI:** 10.3390/ma18122819

**Published:** 2025-06-16

**Authors:** Bogdan Bochenek, Katarzyna Tajs-Zielińska

**Affiliations:** Faculty of Mechanical Engineering, Cracow University of Technology, 31-155 Kraków, Poland; bogdan.bochenek@pk.edu.pl

**Keywords:** structural topology optimization, random loads, cellular automaton mimicking colliding bodies

## Abstract

Topology optimization has been present in modern engineering for several decades, becoming an important tool for solving design problems. Today, it is difficult to imagine progress in engineering design without the search for new approaches to the generation of optimal structural topologies and the development of efficient topological optimization algorithms. The generation of topologies for structures under random loads is one of many research problems where topology optimization is present. It is important to predict the topologies of structures in the case of load uncertainty, since random load changes can significantly affect resulting topologies. This paper proposes an easy-to-implement numerical approach that allows the prediction of the resulting topologies of structures. The basic idea is to transform a random loads case into the deterministic problem of multiple loads. The concept of equivalent load scheme (ELS) is introduced. Instead of generating hundreds of loads applied at random, the selection of a few representative load cases allows the reduction of the numerical effort of the computations. The numerical implementation of proposed concepts is based on the cellular automaton mimicking colliding bodies, which has been recently introduced as an efficient structural topology generator. The examples of topology optimization under randomly applied loads, performed for both plane and spatial structures, have been selected to illustrate the proposed concepts. Confirmed by results of numerical simulations, the efficiency, versatility and ease of implementation of the proposed concept can make an original contribution to research in topological optimization under loads applied in a random manner.

## 1. Introduction

A major advance in the field of engineering optimization occurred in the late 1980s with the publication of the results on topology optimization in the now well-known papers [1,2]. The concept has garnered significant attention and has given rise to a multitude of engineering applications. Every year, new adaptations and algorithms are published as the idea brings wide possibilities. Many review papers provide a comprehensive discussion of various aspects of topological optimization. As examples, refs. [3,4,5,6] may serve, providing a valuable overview of this issue. Topology optimization is developing in a number of directions in all aspects of engineering design. These include the design of systems with consideration of load uncertainty.

A series of approaches and techniques have been advanced for the purpose of dealing with random and uncertain loads [7,8,9,10,11]. The issues of reliability-based topology optimization and robust topology optimization are of particular concern, e.g., in [12,13,14,15,16] and many more, as environmental load variations and various uncertain factors are widely found in engineering practice. Within this area of research, it is worth pointing out a series of recent papers [17,18,19].

As reported in the literature, randomly acting loads generate topologies that differ from those obtained for deterministic loads. The objective of this paper is to present an easy-to-implement, straightforward numerical approach to predicting the resulting topologies of structures under loads that can be applied in a random way. A proposed technique is both simple and effective. The technique is based on transforming random loads into a deterministic multiple load problem. Subsequently, multiple load schemes are represented by a selected number of them. The easy implementation of multiple loads into the optimization procedure has been the motivation to adapt this technique to obtain a topology for loads applied in a random manner.

In particular, in the present paper a case of randomized angle of load application is under consideration. Hence, an angle is randomly selected, and the load applied at this angle is treated as a particular load case. The set of such load cases is then the basis for the topology optimization performed as the multiple load case. It is shown that if the number of load cases taken into account is large enough, the final topology, which agrees with solutions of problems with random loads reported in literature, can be obtained. The new idea proposed in this paper is to take a step forward and to simplify the above-described procedure. The idea is to select a load scheme that involves only a few loads but allows the generation of the same topology of structure as the one obtained under numerous simulated loads applied at random angles and, as a result, the prediction of the topologies, taking into account randomly acting loads, with a simple numerical approach.

One important step in the topology optimization procedure is the selection of an appropriate optimization tool, which is crucial for achieving the desired result. In this paper, the heuristic cellular automaton (CA) method was chosen as the optimization tool due to its simplicity of implementation and its fast convergence properties. Cellular automata are defined as the mathematical idealization of physical systems, where the time and domain are discretized. The cells of the automaton, arranged in a grid, evolve in discrete time steps according to defined local rules, which are also determined by the states of its neighboring cells. The concept proposed by von Neumann and Ulam has attracted significant attention, since it can mimic the complex behavior of the system by applying simple rules of evolution. Cellular automata can be used to model a variety of physical, chemical, biological and social phenomena. In addition to this, cellular automata can also be used as an optimization tool, in particular as an engineering optimization tool. In 1994, the first application of CA in topology optimization was proposed [20]. Following the encouraging results, a number of papers were published in the field, proposing the new rules [21,22,23,24,25] and new applications [26,27,28,29,30]. A number of studies have proven the effectiveness and versatility of the method. A wide range of applications is characteristic for the cellular automata method started from early studies on topology optimization for crashworthiness [31,32] and newer [33,34], including the consideration of large deformation [35], designing of materials with extreme properties [28], multi-scale topology optimization [36], topology optimization of graded multi-material structures [37], optimal design of periodic structures subject to self-weight loading [38], topology optimization of multi-body systems [29], a random vibration topology optimization method and a dynamic-static coupling topology optimization method [39]. This paper deals with the application of the CA method to find optimized structure topologies under random forces. The numerical implementation is based on the cellular automaton mimicking colliding bodies, which has been recently introduced as an efficient structural topology generator.

The paper is structured as follows. In Section 2, the topology optimization problem is formulated, and the algorithm used to solve it is described. The numerical technique to implement random loading in the topology generation process as a set of multiple load cases is presented next, and finally, a new concept of equivalent loading scheme (ELS) is introduced, followed by the introductory example. Section 3 presents illustrative examples of both implemented approaches, with main attention focused on ELS technique. The tasks of topology generation under random loads cover both plane and spatial structures. The application to solve the engineering optimization problem is also presented. Based on the results of the performed tests, the paper ends with some concluding remarks.

## 2. Methods and Concepts

### 2.1. Problem Formulation

Performing topology optimization within a specified structure domain involves generating a material layout that meets the assumed optimality criterion. In practice, some parts of material are relocated, others are selectively removed, and the optimized structure gains a new shape. Over the years, many specific formulations of topology optimization problems have been proposed. The discussion on this subject can be found, for example, in the paper by Lewiński et al. [40]. When formulating structural topology optimization problems, it is very often that the structure compliance is minimized, since minimal compliance results in maximal stiffness of the optimized structure.

While performing structural topology optimization, the finite element-based strategy is one of the most frequently applied. In this paper, the formulation of the problem presented in a widely recognized paper by Sigmund [41] has been utilized, with the objective function and constraints defined within the finite element approach. The compliance c represented by Equation (1) is minimized. The constraint on structure volume *V* is imposed by the available material volume fraction *κ* as defined in Equation (2):

(1) $$\mathrm{minimize}\;c\left(d\right)=u^{T}ku=\sum_{n=1}^{N}d_{n}^{p}u_{n}^{T}k_{n}u_{n}$$

(2) $$\mathrm{subject to}\;V\left(d\right)=\kappa V_{0}$$

(3) ku=f

(4) $$0<d_{min}\leq d_{n}\leq 1.$$

Defined for *N* elements are the quantity $u_{n}$, which represents the displacement vector, and $k_{n}$, which stands for the stiffness matrix. Similarly, the design variable $d_{n}$, which represents the material relative density, is assigned to each element. The global stiffness matrix **k**, global displacement vector **u** and vector of forces **f** form the equation of state Equation (3). To avoid singularity of $d_{n}$, the simple bounds are imposed on the design variables in Equation (4), with $d_{min}$ as a non-zero minimal value of relative density.

As the material representation, the power law SIMP—solid isotropic material with penalization; see, e.g., Bendsoe and Sigmund [42]—is adapted. The modulus of elasticity $E_{n}$ assigned to each finite element is a function of the design variable $d_{n},$ whereas the quantity $E_{0}$ stands for the modulus of elasticity representing solid material:

(5) $$E_{n}=d_{n}^{p}E_{0}.$$

In Equation (5), p (typically p = 3) is responsible for penalization of intermediate densities and, by controlling the design process that way, allows the obtaining of black-and-white resulting structures. Removing unnecessary parts from a design criterion point of view leads to a redistribution of material within the design domain, which results in a new structure topology.

### 2.2. Topology Optimization Algorithm

The algorithm built as a cellular automaton is proposed to solve the problem formulated in this paper. Its performance is based on specially selected local update rules, which are responsible for performing the optimal topology generation process. The design domain is decomposed into a uniform lattice of cells, usually equivalent to finite elements, and the interaction between cells takes place only within the cell neighborhood. According to the recent proposal [23], the efficient rules can be built so as to mimic the colliding bodies phenomenon. The cells of the automaton collide locally with the neighboring cells, which results in pushing them away or remaining in their positions. As a result, the material is redistributed within the design domain and at the end of this process a new topology is created.

The proposed update rule takes the form of Equation (6):

(6) $$d_{n}^{new}=d_{n}+F_{n}^{AVG}m,$$

where $F_{n}^{AVG}$ denotes design variable $d_{n}$ correction component, and m stands for a move limit (e.g., m = 0.2). The form of the $F_{n}^{AVG}$,

(7) $$F_{n}^{AVG}=\frac{1}{M}\sum_{k=1}^{M}\frac{{(A}_{k}-A_{n})F_{n}+2A_{k}F_{k}}{A_{n}+A_{k}}$$

has been derived based on simulation of collisions of a cell n with its k=1,2…M neighbors, as described in [23]. In Equation (7) $A_{n},A_{k}$ are the cell areas, whereas values of $F_{n},F_{k}$ are calculated based on local compliance values for the central and neighboring cell, respectively. Based on the results of the structural analysis, the values of local compliances are calculated for all cells/elements and then sorted in ascending order. Those having the lowest and the highest values are identified, $N_{1},N_{2}$ are selected, and values of $F_{n}$ are assigned according to Equation (8):

(8) $$F_{n}=\left\{\begin{array}{cc} -C & n<N_{1}\\ f_{n} & N_{1}\leq n\leq N_{2}\\ C & n>N_{2} \end{array}\right.$$

where, for the intermediate interval $N_{1}\leq n\leq N_{2},$ a monotonically increasing function representing elements’ compliances is selected:

(9) $$f_{n}=2C\frac{n}{N_{2}-N_{1}}-C\frac{N_{2}+N_{1}}{N_{2}-N_{1}}.$$

The user-specified parameter C usually equals 1.

In the iterative optimization procedure, the structural analysis performed for the optimized element is followed by a local updating process. At the same time, a global volume constraint is applied, and as a result, the generated topologies preserve a specified volume fraction of a solid material during the iterative process.

### 2.3. Multiple Load Case in Topology Optimization and the Concept of the Paper

**Figure 1:**
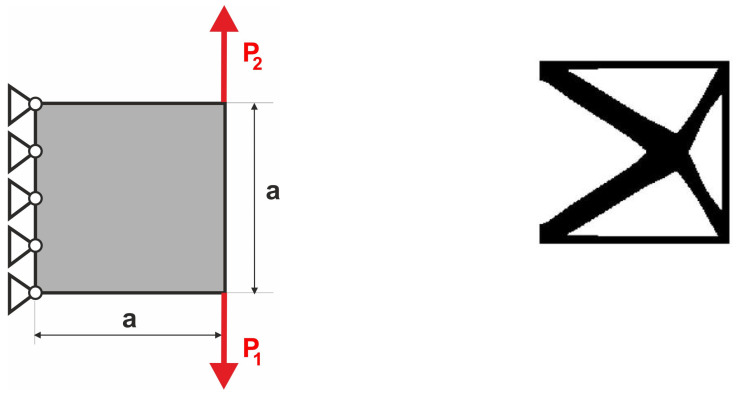
The structure under the two-load case P_1_, P_2_ (**left**). The resulting topology (**right**).

The compliance minimization problem formulated above can easily be adapted to a multiple load case. This was pointed out, among others, in papers by Bendsoe [43], Bendsoe and Sigmund [42] and Sigmund [41]. Like for multi-objective optimization problems, by using the weighted sum of compliances subjected to all considered load cases, multiple loads can be included in the search for the optimal structural topology. As a result, only a slight modification of the original topology algorithm is necessary to cope with this problem. Below, the illustrative example of the two-load case previously discussed in [41] is recalled; see Figure 1.

In order to solve this problem, the structural analysis was performed for both load cases, and the objective was defined as the sum of compliances found for each case. The only modification within the optimization algorithm required replacement of a single compliance by the sum of compliances $c=c^{\left(1\right)}+c^{\left(2\right)}$.

**Figure 2:**
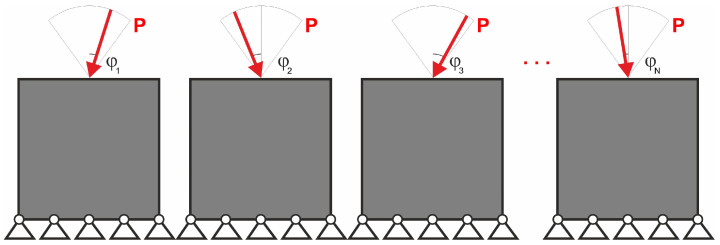
Multiple load case mimicking load applied at randomly selected angles.

The easy implementation of the multiple load case into the topology optimization problem was the inspiration to adapt this to deal with loads applied in a random way. In what follows, a set of loads for which positions or angles of application are generated as random quantities may be treated as a set of particular load cases. This idea is illustrated by Figure 2, below.

**Figure 3:**
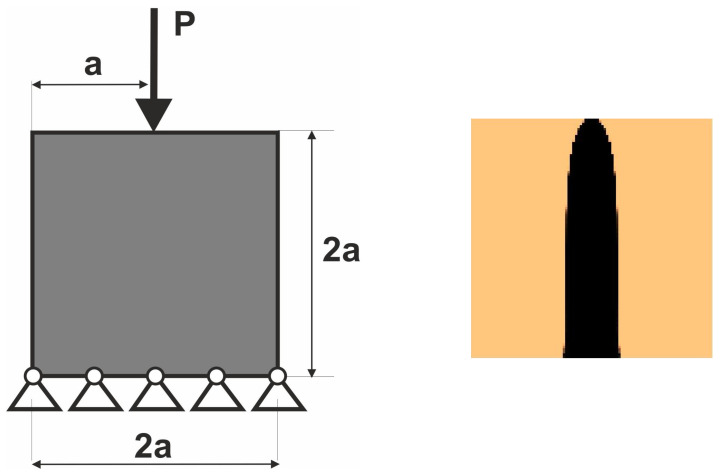
The structure under a single load—deterministic case (**left**). The structure topology for the volume fraction κ  = 0.2 (**right**).

The set of such load cases is then the basis for the topology optimization performed as the multiple load case. This idea was presented and illustrated in [44]. Below (Figure 3), a simple example of this concept implementation is presented.

The random change of load application angle has been allowed according to $\varphi^{\left(i\right)}=\left(2r-1\right)\alpha ,$ where r is a random number taken from uniform distribution. This means that a random angle φ varies from −α to α . The vertical and horizontal load components are then implemented as Pcos φ and Psin φ, respectively. The regular mesh of 10,000 (100 × 100) elements/cells has been applied.

The important observation, based on the results of numerical simulations, is that the number of load cases need to mimic load acting as random can be reasonably low. Moreover, as compared to deterministic solutions, the generated topologies represent the same layout changes as those reported in the literature. The discussion in [44] has been limited to plane structures.

This paper proposes two extensions based on the above concept. The first one is to broaden the discussion to include spatial structures, and this is accomplished in Section 3.2 of the paper. As the second extension, an alternative approach to dealing with random angle of load application is proposed. The details are included in the following section.

### 2.4. Equivalent Load Scheme (ELS) and Its Implementation

It has been shown that the sets of loads applied at randomly selected angles treated as the multiple load case, combined with the topology algorithm’s ability to deal with such problems, allow generation of minimal compliance topologies. Nevertheless, the number of loads needed to mimic a load acting as random, even if reasonable, usually causes time-consuming computations, especially for the spatial structures. It is worth remembering that each load case requires structural analysis to be performed. The idea is therefore to select a load scheme which involves only a few loads but allows the generation of the same topology of structure as the one obtained under simulated loads applied at random angles. This leads to the proposal of the **equivalent load scheme—ELS**.

**Figure 4:**
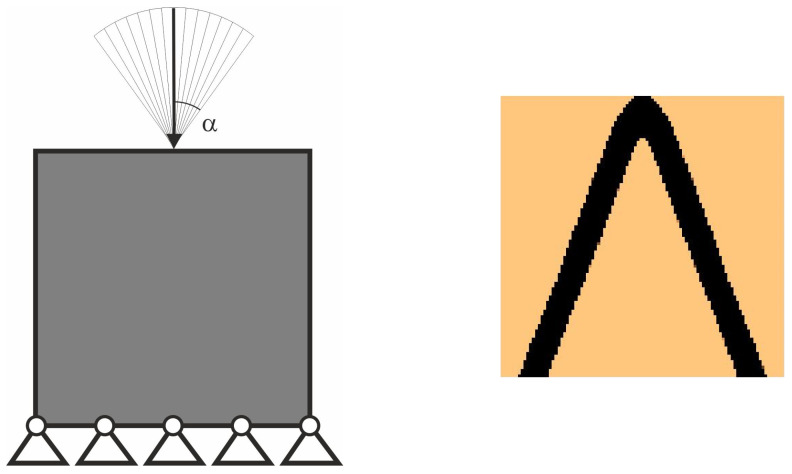
The structure under a load whose angle of application has been randomly selected from [−α, α] (**left**). The structure topology for 100 loads case, α = π/9 (**right**).

**Figure 5:**
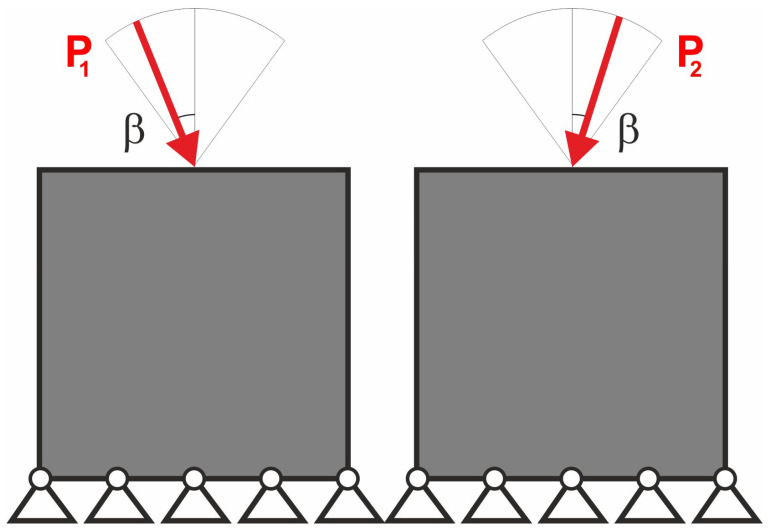
The 2-load equivalent load scheme (ELS).

Let us see the illustrative example of this concept. The structure under random loads from Figure 4 is considered. Instead of a set of loads with random angle of load application *φ* taken from −α to α, the equivalent load scheme (ELS) is proposed here in the form of two loads acting at the angle β = α/2; see Figure 5. The value α/2 has been selected because angle *φ* has been randomly selected from the uniform distribution.

**Figure 6:**
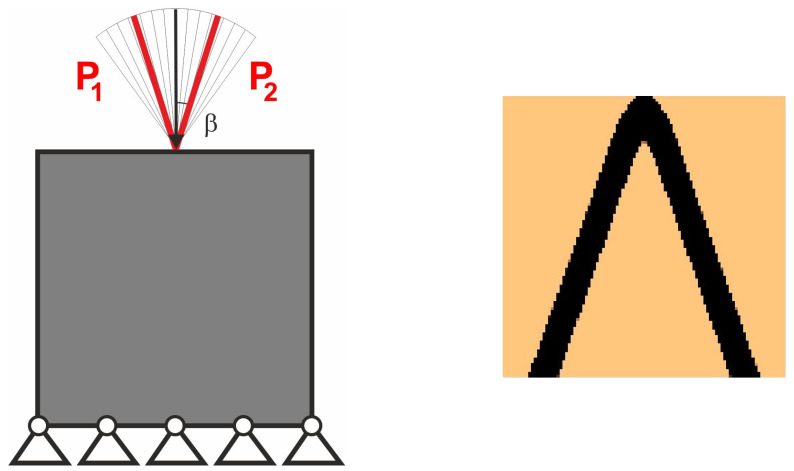
The structure under the equivalent load scheme (ELS) = [P_1_, P_2_] (**left**). The structure topology for the 2-load equivalent load scheme (ELS), β = π/18 (**right**).

The topology has been generated for the equivalent load scheme, and the result is presented in Figure 6. One can observe that the topologies presented in Figure 4 and Figure 6 are no different. The deterministic equivalent load scheme (ELS) allows the prediction of the topology of the structure under loads applied at random angles.

## 3. Results and Discussion

With a view to illustrate the concept proposed in this paper, some test problems are solved. The discussed tasks include both plane and spatial structures, among which is an engineering structure. The Matlab-based topology algorithm has been utilized in the case of the plane test structures. While dealing with the spatial structures, the topology generator has been combined with the ANSYS 2025 R1 system, which performs the structural analysis.

Topologies are generated for the three cases, namely the structure under deterministic load, the structure under a set of loads with random angle of load application and the structure under the equivalent load scheme (ELS).

### 3.1. Generation of Plane Topologies Under Loads Applied at Random Angles

#### 3.1.1. Plane Structure 1

**Figure 7:**
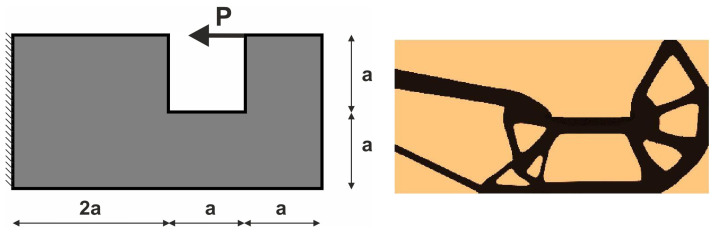
Structure 1 under a single deterministic load (**left**) and its final topology (**right**).

For the structure shown in Figure 7, the mesh of 400 × 200 (80,000) square elements/cells has been implemented. The structural analysis and topology generation for the volume fraction *κ* = 0.3 have been performed, for $E_{0}\;$= 10 GPa, *ν* = 0.3, *P* = 100 N, a = 100 mm. The Moore-type neighborhood has been applied. This structure was selected as one of the test structures in [23].

**Figure 8:**
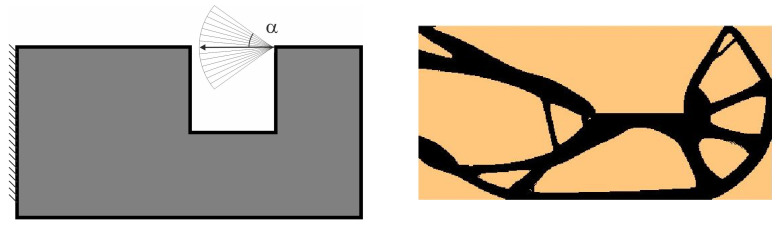
Structure 1 under a load whose angle of application has been randomly selected from [−α, α] (**left**) and its final topology for 100 loads case, α = π/6 (**right**).

**Figure 9:**
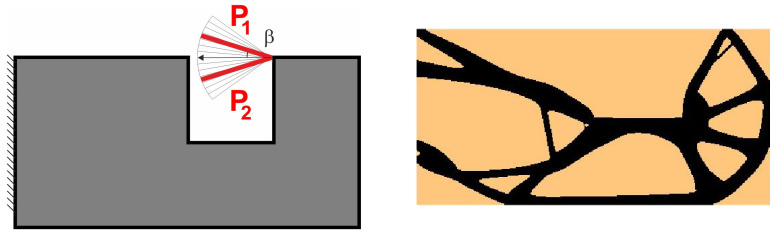
Structure 1 under the equivalent load scheme (ELS) = [P_1_, P_2_] (**left**). The structure topology for the 2-load ELS case, β = π/12 (**right**).

The angle of load application is treated as a random value generated according to the uniform distribution (Figure 8). The deterministic equivalent load scheme (ELS) is presented in Figure 9.

#### 3.1.2. Plane Structure 2

**Figure 10:**
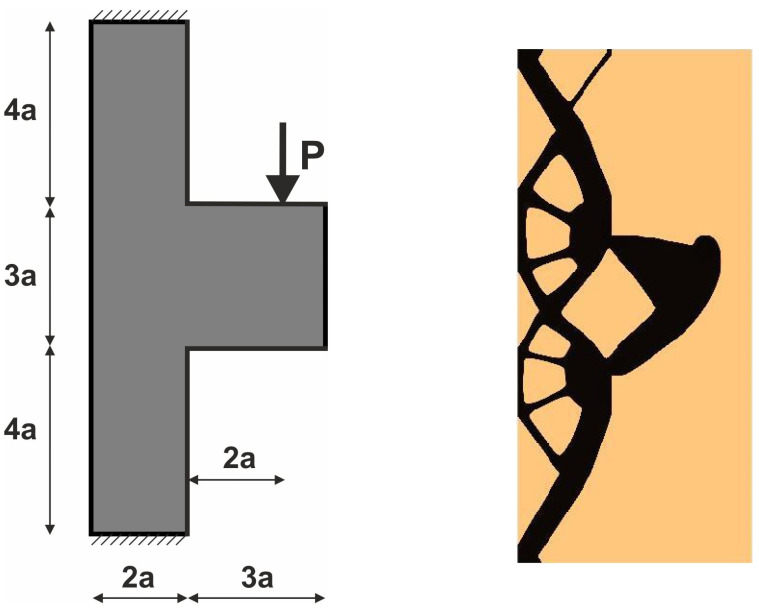
Structure 2 under a single deterministic load (**left**) and its final topology (**right**).

**Figure 11:**
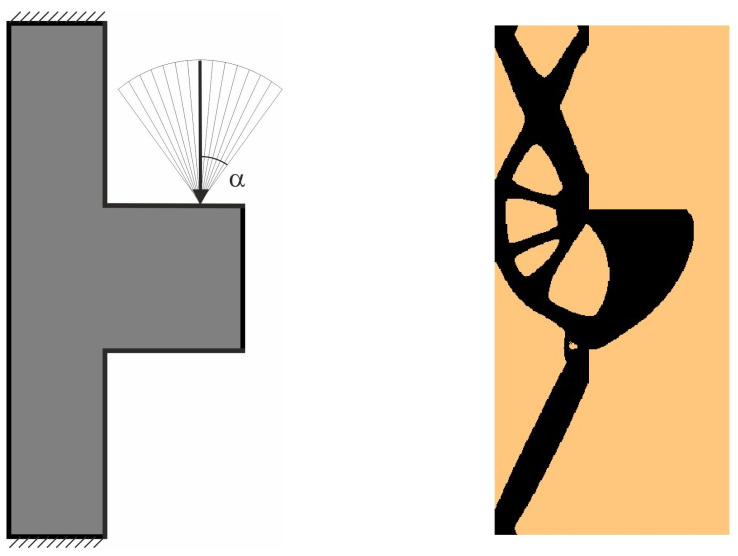
Structure 2 under a load whose angle of application has been randomly selected from [−α, α] (**left**) and its final topology for 100 loads case, α = π/4 (**right**).

**Figure 12:**
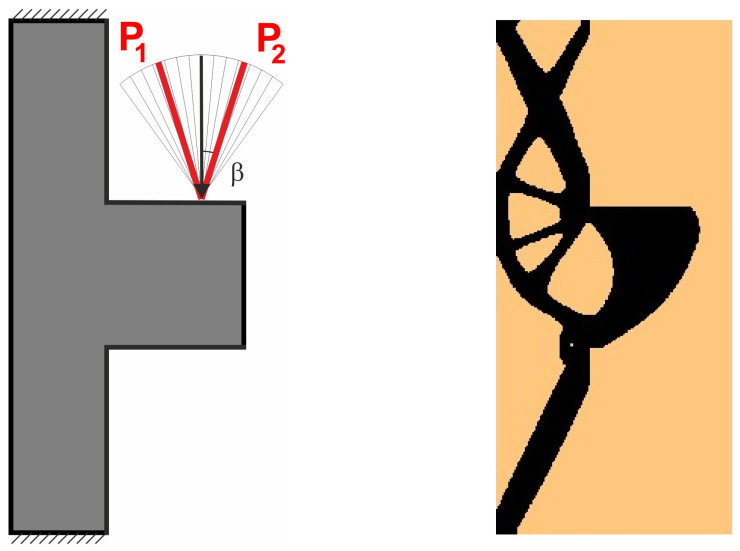
Structure 2 under the equivalent load scheme (ELS) = [P_1_, P_2_] (**left**). The structure topology for the 2-load ELS case, β = π/8 (**right**).

For the structure shown in Figure 10, the mesh of 250 × 550 (137,500) square elements/cells has been implemented. The structural analysis and topology generation for the volume fraction *κ* = 0.25 have been performed, for $E_{0}\;$= 10 GPa, *ν* = 0.3, *P* = 100 N, a = 50 mm. The deterministic solution for this structure was presented as one of the test structures in [23]. Figure 11 and Figure 12 show the structure 2 under a load whose angle of application has been randomly selected from [−α, α] and the structure 2 under the deterministic equivalent load scheme (ELS) including the final topologies.

#### 3.1.3. Plane Structure 3

**Figure 13:**
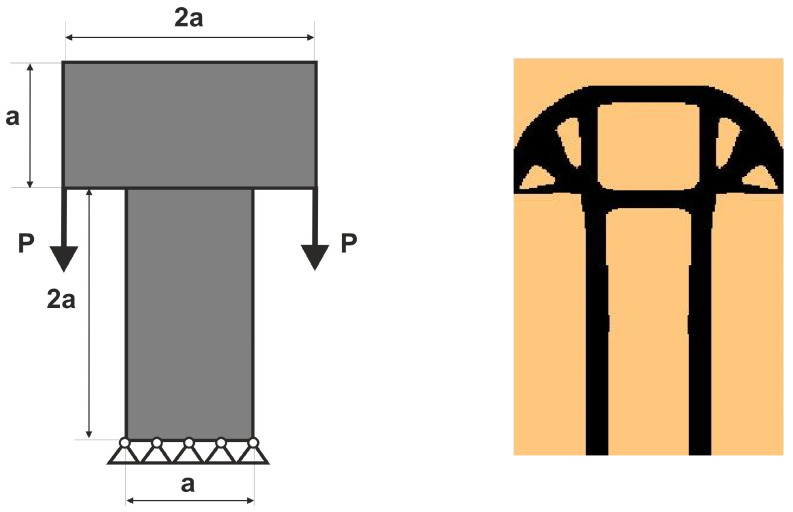
Structure 3 under a single deterministic load (**left**) and its final topology (**right**).

For the tower structure shown in Figure 13, the mesh of 160 × 240 (38,400) square elements/cells has been implemented. The structural analysis and topology generation for the volume fraction *κ* = 0.25 have been performed, for $E_{0}\;$= 1 MPa, *ν* = 0.3, *P* = 1 N, a = 80 mm.

**Figure 14:**
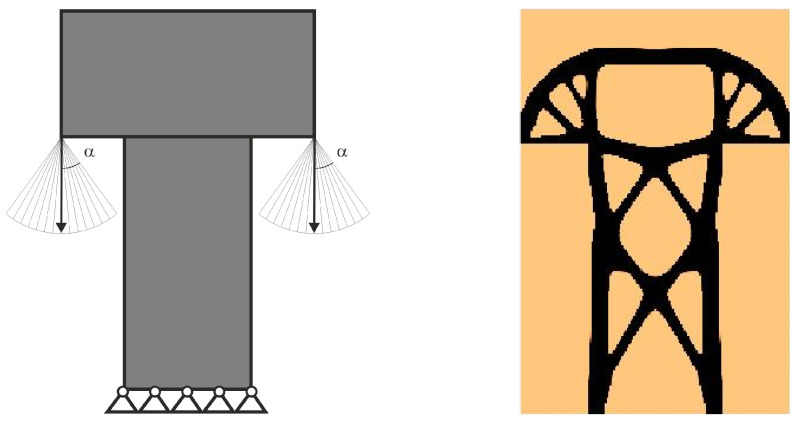
Structure 3 under a load whose angle of application has been randomly selected from [−α, α] (**left**) and its final topology for 100 loads case, α = π/4 (**right**).

**Figure 15:**
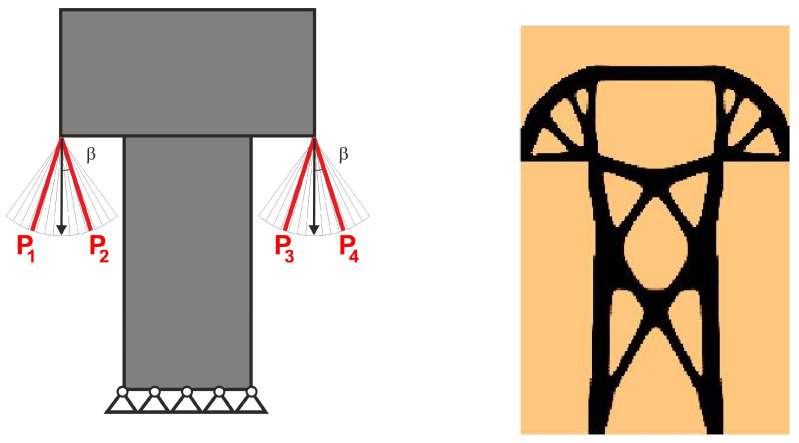
Structure 3 under the equivalent load scheme (ELS) = [P_1_ + P_4_, P_2_ + P_3_, P_1_ + P_3_, P_2_ + P_4_] (**left**). The structure topology for the 4-load ELS case, β = π/8 (**right**).

The angle of applied load, see Figure 14, is treated as the random value taken from interval −α to α according to uniform distribution. This structure was selected as one of the test structures in [44], and the topology of this structure under random loads was presented there. Figure 14 and Figure 15 show structures 3 under loads with randomly selected angles of application within the range [−α, α], and under the deterministic equivalent load scheme (ELS), including the final topologies.

### 3.2. Generation of Spatial Topologies Under Loads Applied at Random Angles

#### 3.2.1. Spatial Structure 1

The proposed idea was tested for selected three-dimensional examples. A significant benefit of the method is its capacity for straightforward integration with any finite element analysis tool. The integration with Ansys facilitates the analysis of complicated geometries with a uniform or non-uniform mesh of finite elements, which allows for optimization of realistic engineering structures. Consequently, the optimization process for the three-dimensional example is based on structural analysis performed by the Ansys Mechanical tool, and the updating process is based on the presented cellular automata rules.

**Figure 16:**
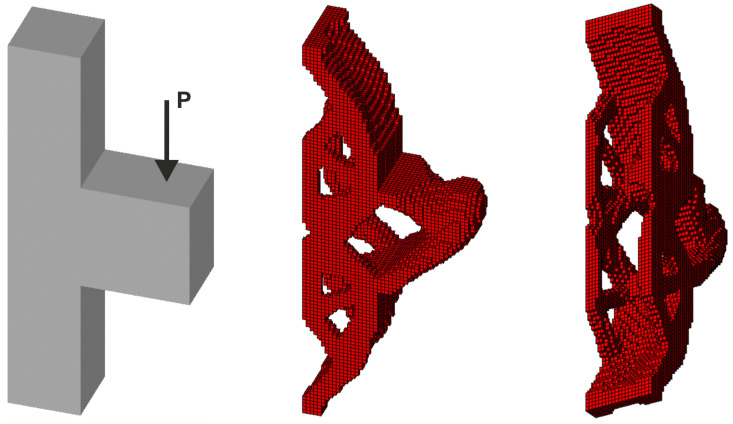
The structure under a single deterministic load (**left**) and its final topology—two perspectives (**right**).

The first three-dimensional test structure is plane test structure 3, defined now with a thickness of 2a; see Figure 16 (left). At the first step, the final topology for a single deterministic vertical load *P* = 100 N has been found as it is presented in Figure 16 (right) for $E_{0}\;$= 10 GPa, *ν* = 0.35 and *κ* = 0.3.

**Figure 17:**
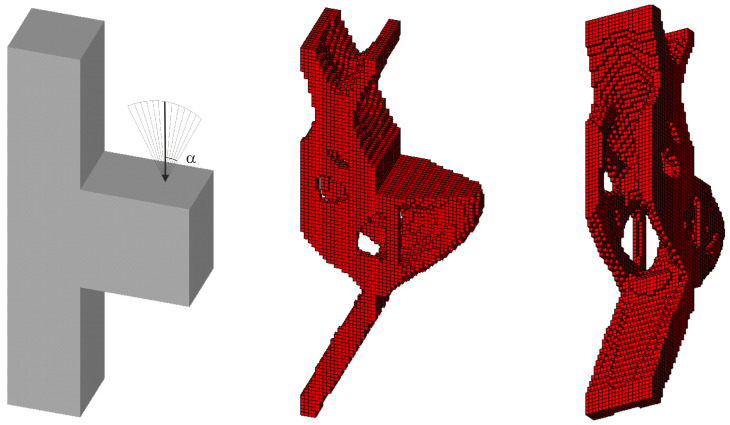
The structure under a load whose angle of application has been randomly selected from [−α, α] (**left**) and its final topology—two perspectives, α = π/6 (**right**).

Next, 100 loads, acting in one plane, have been applied at angles randomly selected from the interval [−π/6, π/6]; see Figure 17.

**Figure 18:**
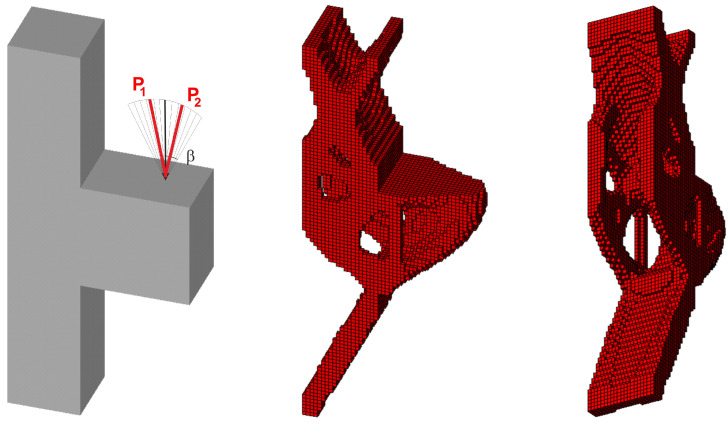
The structure under the equivalent load scheme (ELS) = [P_1_, P_2_] (**left**). The structure topology for the 2-load ELS case, β = π/12—two perspectives (**right**).

This set of forces can be effectively replaced by the equivalent load scheme (ELS), for which only two forces acting at the angle β = α/2 = π/12 are utilized in this instance. As is clearly evident, the resulting topology is almost identical (see Figure 18).

#### 3.2.2. Spatial Structure 2

**Figure 19:**
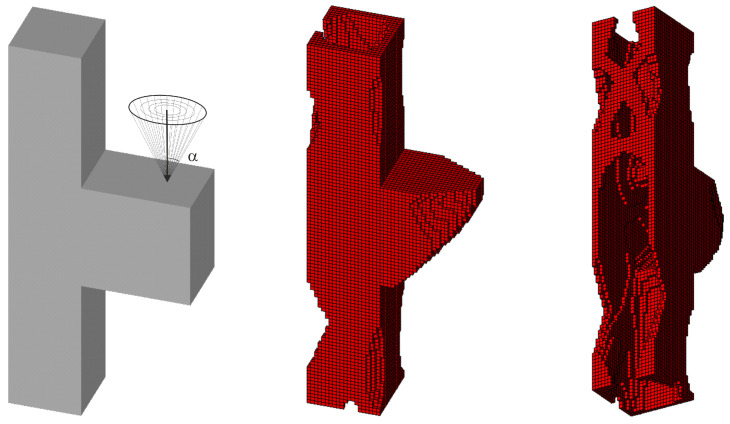
The structure under a load whose angles of application have been randomly selected from [−α, α] and [−π, π] for vertical and horizontal planes, respectively (**left**), and its final topology for 100 loads case, α = π/6—two perspectives (**right**).

Furthermore, the task under consideration has been expanded to the example of random forces acting in both planes within the range of variation of the angle α as previously defined (see Figure 19). The random change of load application angle has been allowed for according to $\varphi^{\left(i\right)}=\left(2r-1\right)\alpha$ and $\psi^{\left(i\right)}=\left(2r-1\right)\pi$, where r is a random number taken from uniform distribution. This means that the random angle φ varies from −α to α, whereas angle ψ varies from −π to π. The vertical and horizontal load components are then implemented as Pcos φ,Psin φcos ψ and Psin φsin ψ, respectively.

**Figure 20:**
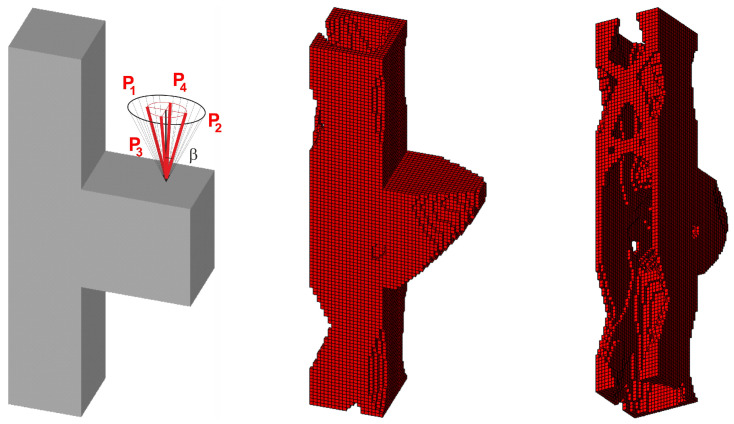
The structure under the equivalent load scheme (ELS) = [P_1_, P_2_, P_3_, P_4_] (**left**). The structure topology for the 4-load ELS case, β = π/12—two perspectives (**right**).

The set of random loads is now replaced by the equivalent load scheme (ELS) of four deterministic loads. The obtained final topologies can be compared in Figure 19 and Figure 20.

#### 3.2.3. Spatial Structure 3: Engineering Design

**Figure 21:**
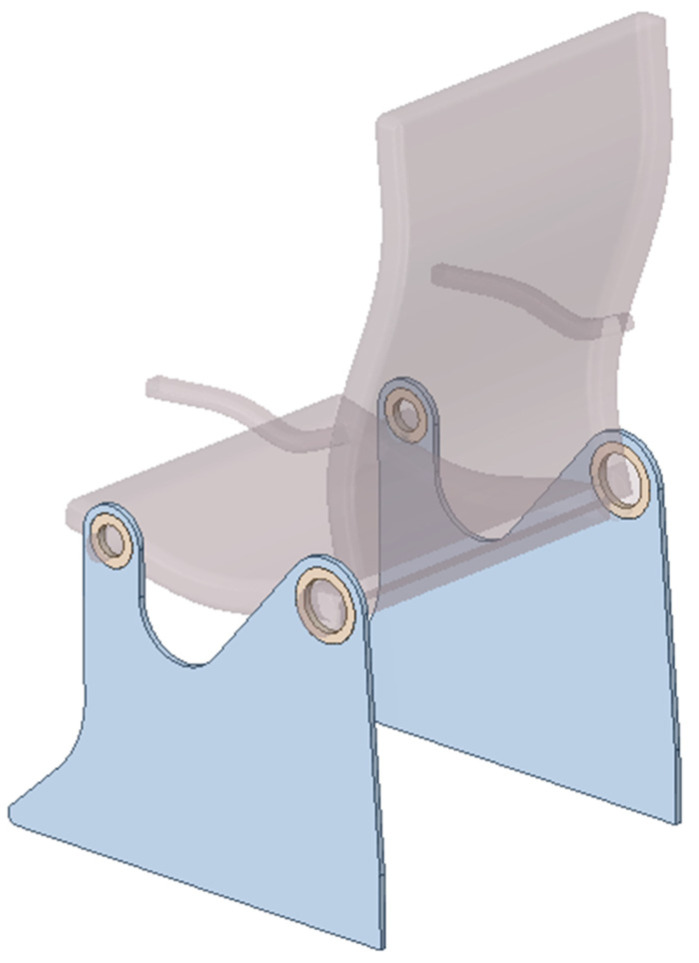
Initial model of the aircraft seat leg; design space marked in blue.

**Figure 22:**
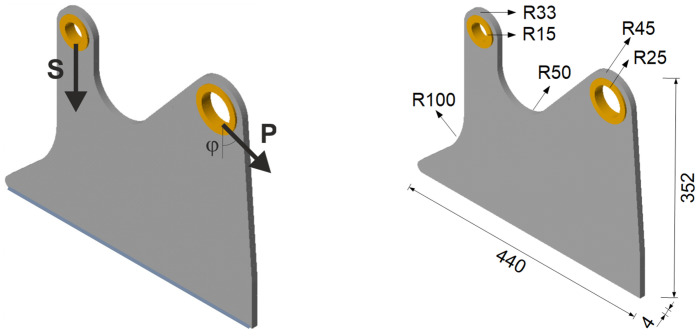
The aircraft seat leg structure under a single deterministic load (**left**) and its dimensions (**right**).

The proposed technique is then used to determine the minimal compliance topology of the aircraft seat leg, shown in blue in Figure 21, loaded by forces acting at the right mounting hole, as shown in Figure 22. The vertical load in the left mounting hole models the weight of a passenger (2400 N) and is considered as a deterministic one.

This study is inspired by [45], where the multi-material topology optimization of a similar structure is presented. The choice seems to be rational, considering that forces acting on the backrest are non-deterministic when the chair is in use. To simplify, dynamic loads have been omitted; see [46].

The bottom edge of the structure is fixed. The non-optimized regions are shown in Figure 22 as the yellow volumes of mounting rings. The dimensions of the structure are shown in Figure 22 (right), while the material data are as follows: $E_{0}\;$= 210 GPa, ν = 0.35, and the assumed volume fraction is *κ* = 0.25.

**Figure 23:**
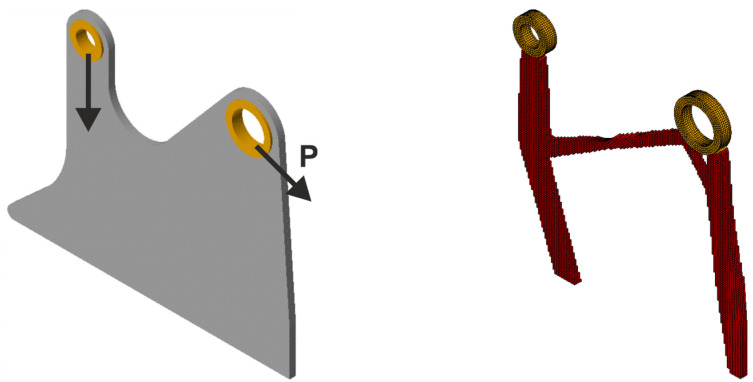
The structure under a deterministic load (**left**). The final topology (**right**).

In the first step of the analysis, the deterministic load P = 2000N is applied for φ = π/4. The initial design domain with the scheme of acting load and the final topology for this case are shown in Figure 23.

**Figure 24:**
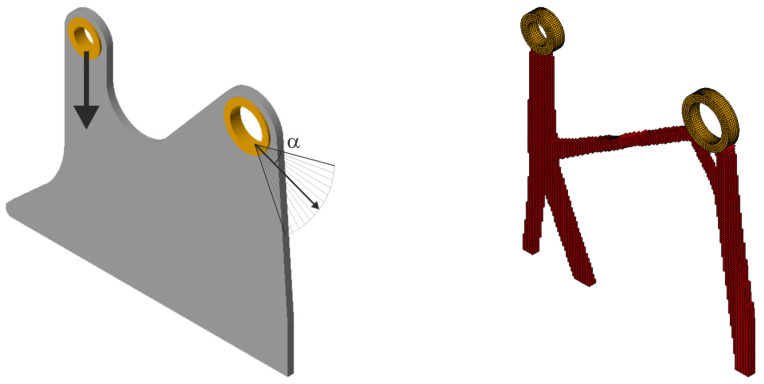
The structure under a load whose angle of application has been randomly selected from [−α, α] (**left**) and its final topology for 100 loads case, α = π/6—two perspectives (**right**).

**Figure 25:**
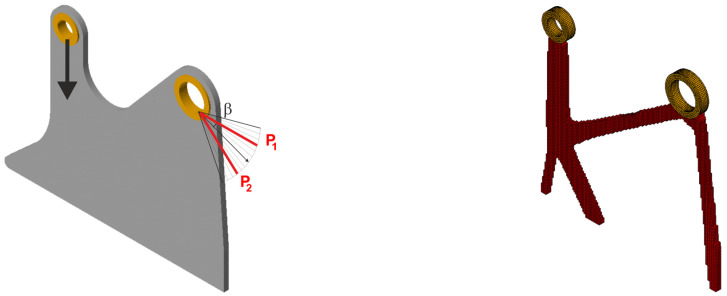
The structure under the equivalent load scheme (ELS) = [P_1_, P_2_] (**left**). The structure topology for the 2-load ELS case, β = π/12 (**right**).

Next, 100 loads, acting in one plane, have been applied at angles randomly selected from the interval [−π/6, π/6]; see Figure 24 (left). The forces of this set can be effectively replaced by a limited number of loads—in this case, only two; see Figure 25 (left). The resulting topology is almost identical for Figure 24 (right) and 25 (right).

**Figure 26:**
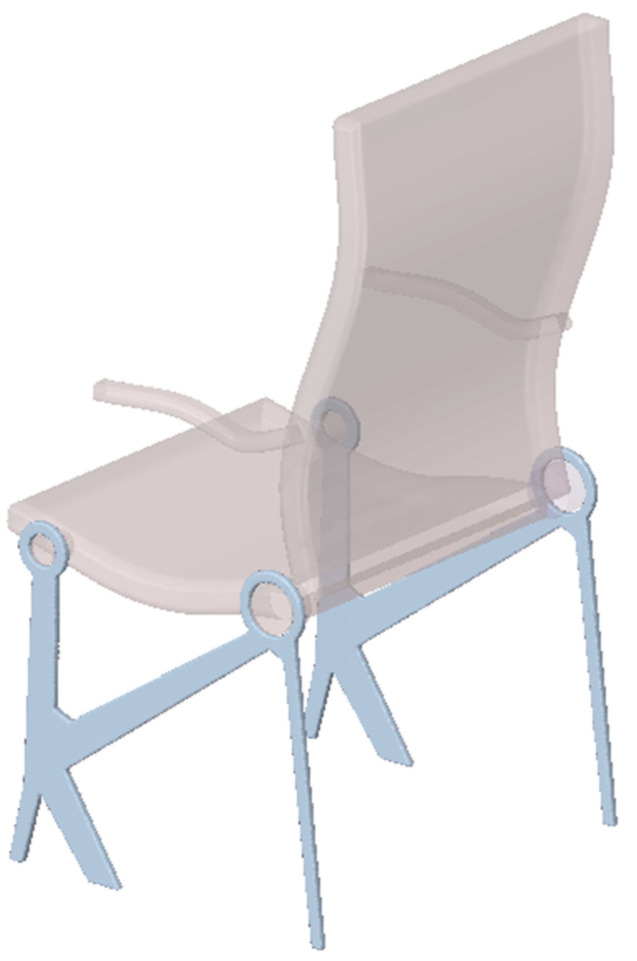
Final construction of aircraft seat leg—visualization.

The engineering visualization of the resulting topology is shown in Figure 26.

The proposed approach to modeling topologies for structures subjected to random loads is illustrated by minimizing the compliance of linear, elastic, one-material structures subject to a total volume constraint. In this formulation of the topology optimization problem, the selection of the material’s Young modulus does not influence the final topology. The value of the applied load also has no influence on the final topology. As for the Poisson ratio, its small influence can be observed, even though it is usually omitted in papers on topology optimization.

### 3.3. Illustration of the ELS Concept Versatility

**Figure 27:**
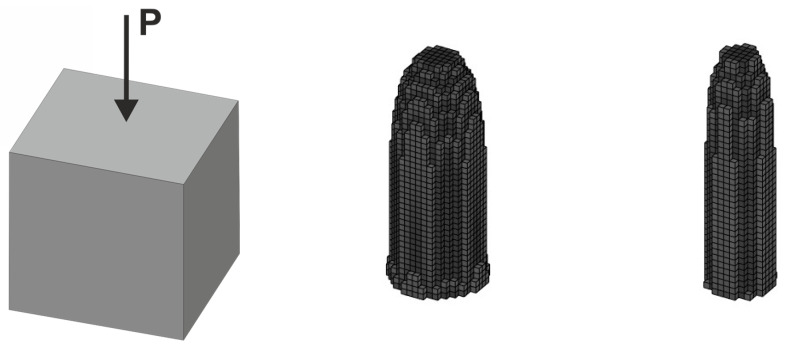
The cube structure (40 × 40 × 40 elements) under a single deterministic load (**left**) and its final topologies obtained for two volume fractions: 0.10 and 0.05 (**right**).

As shown in former sections, the ELS concept allows the effective prediction of the structure topology in the case of a load applied at a random angle. It is also worth pointing out that this approach is a versatile one and can be implemented in any algorithm suited for compliance minimization. As an example, the algorithm introduced in [47] has been adapted to solve the classic cube problem. The cube structure is shown in Figure 27. Similarly to other tasks discussed in the paper, the deterministic problem with a single load has been solved first, and then the random change of the load angle application has been taken into account.

**Figure 28:**
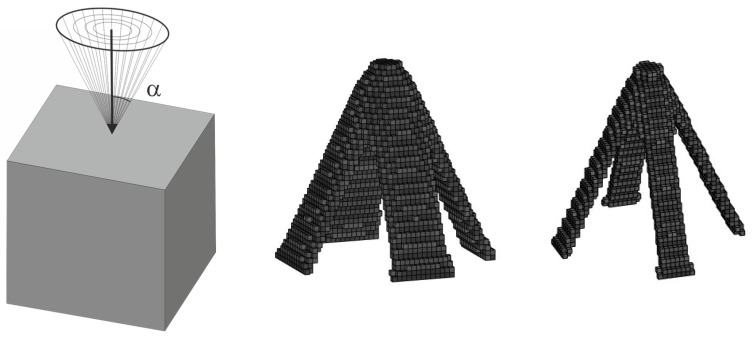
The cube structure (40 × 40 × 40 elements) under a load whose angles of application have been randomly selected from [−α, α] and [−π, π] for vertical and horizontal planes, respectively (**left**), and its final topology for 200 loads case, α = π/5—two volume fractions: 0.10 and 0.05 (**right**).

In what follows, a set of 200 load cases with randomly generated angles of load application has been considered, which has resulted in the topology presented in Figure 28 for two volume fractions of resulting topologies.

**Figure 29:**
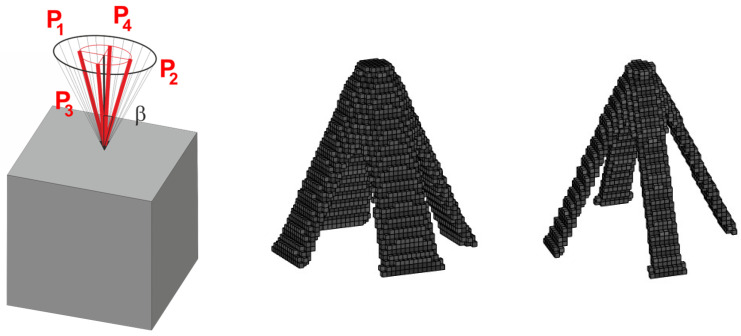
The cube structure (40 × 40 × 40 elements) under the equivalent load scheme (ELS) = [P_1_, P_2_, P_3_, P_4_] for β=π/10 (**left**) and its final topologies obtained for two volume fractions: 0.10 and 0.05 (**right**).

Then, ELS approach has been applied with a four-load case, as shown in Figure 29. One can observe that the resulting topologies, obtained under a set of 200 load cases with randomly generated angles of load application and using the ELS approach, duplicate each other.

## 4. Conclusions

Summarizing the results and discussion presented in the paper, some conclusions can be made. First of all, it has been shown that the easy-to-implement idea of transforming the load acting as random into the deterministic problem of multiple loads can be an efficient tool to deal with structural topology generation under random loads. This recently introduced concept successfully applied to plane structures has been expanded, in the present paper, to include spatial structural elements.

The new idea proposed in this paper is to take a step forward and to simplify the above-described procedure. The idea is to select a load scheme that involves only a few loads but allows the generation of the same topology of structure as the one obtained under numerous simulated loads applied at random angles. This leads to the proposal of the equivalent load scheme—ELS. A reasonably small number of load cases can mimic the load acting as random and is sufficient to predict the final topology. Instead of generating hundreds of random loads, the selection of a few representative load cases allows the reduction of the numerical effort of the computations.

It is also worth pointing out that this approach is a versatile one and can be implemented in any algorithm suited for compliance minimization. Another benefit is that it can be combined with any structural analysis system, as presented in the example of the Ansys system implementation.

Although the presented concept of equivalent load scheme (ELS) can be applied to any topology optimization method, the numerical implementation of the proposed concepts is based on the cellular automaton mimicking colliding bodies, which has recently been introduced as an efficient structural topology generator. The advantage of using this algorithm, besides the possibility of obtaining fine optimal topologies, is also that the so-called grey areas can be eliminated without using any additional filtering.

Confirmed by the results of numerical simulations, the efficiency and ease of implementation of the proposed concepts can make an original contribution to the research in topological optimization under random loads.

## Data Availability

The original contributions presented in this study are included in the article. Further inquiries can be directed to the corresponding author.

## References

[1] Bendsoe M.B., Kikuchi N. (1988). Generating optimal topologies in optimal design using a homogenization method. Comput. Methods Appl. Mech. Eng..

[2] Bendsoe M.P. (1989). Optimal shape design as a material distribution problem. Struct. Multidiscip. Optim..

[3] Tang T., Wang L., Zhu M., Zhang H., Dong J., Yue W., Xia H. (2024). Topology Optimization: A Review for Structural Designs Under Statics Problems. Materials.

[4] Ribeiro T.P., Bernardo L.F.A., Andrade J.M.A. (2021). Topology Optimisation in Structural Steel Design for Additive Manufacturing. Appl. Sci..

[5] Logo J., Ismail H. (2020). Milestones in the 150-year history of topology optimization: A review. Comput. Assist. Methods Eng. Sci..

[6] Eschenauer H.A., Olhoff N. (2001). Topology optimization of continuum structures: A review. Appl. Mech. Rev..

[7] Csebfalvi A. (2017). Robust topology optimization: A new algorithm for volume-constrained expected compliance minimization with probabilistic loading directions using exact analytical objective and gradient. Period. Polytech.-Civ. Eng..

[8] Martínez-Frutos J., Herrero-Pérez D. (2018). Evolutionary topology optimization of continuum structures under uncertainty using sensitivity analysis and smooth boundary representation. Comput. Struct..

[9] Maute K., Rozvany G.I.N., Lewiński T. (2014). Topology Optimization under Uncertainty. Topology Optimization in Structural and Continuum Mechanics. CISM International Centre for Mechanical Sciences.

[10] De S., Hampton J., Maute K., Doostan A. (2020). Topology optimization under uncertainty using a stochastic gradient-based approach. Struct. Multidiscip. Optim..

[11] Luo K., He X., Jing H. (2024). Topology optimization of bridges under random traffic loading using stochastic reduced-order models. Probabilist. Eng. Mech..

[12] Yin F., Dang K., Yang W., Ding Y., Xie P. (2021). An efficient approach to reliability-based topology optimization for the structural lightweight design of planar continuum structures. J. Mech..

[13] Tauzowski P., Blachowski B., Logo J. (2021). Topology optimization of elasto-plastic structures under reability constraints: A first order approach. Comput. Struct..

[14] Beyer H.G., Sendhoff B. (2007). Robust optimization—A comprehensive survey. Comput. Methods Appl. Mech. Eng..

[15] Jalalpour M., Tootkaboni M. (2016). An efficient approach to reliability-based topology optimization for continua under material uncertainty. Struct. Multidiscip. Optim..

[16] Liu J., Gea H.C. (2018). Robust topology optimization under multiple independent unknown-but-bounded loads. Comput. Methods Appl. Mech. Eng..

[17] Li Z., Wang L., Xinyu G. (2024). A level set reliability-based topology optimization (LS-RBTO) method considering sensitivity mapping and multi-source interval uncertainties. Comput. Methods Appl. Mech. Eng..

[18] Li Z., Wang L., Gu K. (2024). Efficient reliability-based concurrent topology optimization method under PID-driven sequential decoupling framework. Thin-Walled Struct..

[19] Li Z., Wang L., Chai Y., Zhang L., Liu Y. (2025). Topology design of multi-scale thermo-elastic structures considering performance reduction and disturbance based on interval models. Appl. Math. Model..

[20] Inou N., Shimotai N., Uesugi T. A cellular automaton generating topological structures. Proceedings of the 2nd European Conference on Smart Structures and Materials.

[21] Tovar A., Patel M.N., Niebur G.L., Sen M., Renaud J.E. (2006). Topology Optimization Using a Hybrid Cellular Automaton Method With Local Control Rules. J. Mech. Des..

[22] Tajs-Zielińska K., Bochenek B. (2021). Topology algorithm built as an automaton with flexible rules. Bull. Pol. Acad. Sci..

[23] Bochenek B., Tajs-Zielinska K. (2022). Cellular Automaton Mimicking Colliding Bodies for Topology Optimization. Materials.

[24] Deng X., Chen H., Xu Q., Feng F., Chen X., Lv X., Lin X., Fu T. (2022). Topology optimization design of three-dimensional multi-material and multi-body structure based on irregular cellular hybrid cellular automata method. Sci. Rep..

[25] Kita E., Toyoda T. (2000). Structural design using cellular automata. Struct. Multidiscip. Optim..

[26] Tajs-Zielińska K., Bochenek B. (2020). CARMA—Cellular Automata with Refined Mesh Adaptation—The Easy Way of Generation of Structural Topologies. Appl. Sci..

[27] Guan W., Xu G., Kou L., Li W., Liu H., Xu L., Wang X. (2025). A hybrid cellular automata-based topology optimization method for incompressible fluid flow channels. Flow Meas. Instrum..

[28] Da D.C., Chen J.H., Cui X.Y., Li G.Y. (2017). Design of materials using hybrid cellular automata. Struct. Multidiscip. Optim..

[29] Mendoza-Cuy A., Begambre-Carrillo O., Villalba-Morales J.D. (2025). Topology optimization of steel slotted dampers with the hybrid cellular automata technique. Adv. Eng. Softw..

[30] Goetz J., Tan H., Renaud J., Tovar A. (2012). Two-material optimization of plate armor for blast mitigation using hybrid cellular automata. Eng. Optim..

[31] Patel N., Kang B.S., Renaud J.E., Tovar A. (2009). Crashworthiness Design Using Topology Optimization. J. Mech. Des..

[32] Duddeck F., Hunkeler S., Lozano P., Wehrle E., Zeng D. (2016). Topology Optimization for Crashworthiness of Thin-Walled Structures Under Axial Impact Using Hybrid Cellular Automata. Struct. Multidiscip. Optim..

[33] Afrousheh M., Marzbanrad J., Gohlich D. (2019). Topology optimization of energy absorbers under crashworthiness using modified hybrid cellular automata (MHCA) algorithm. Struct. Multidiscip. Optim..

[34] Aulig N., Nutwell E., Menze L.S., Detwiler D. (2018). Preference-based topology optimization for vehicle concept design with concurrent static and crash load cases. Struct. Multidiscip. Optim..

[35] Guo L.S., Tovar A., Penninger C.L., Renaud J.E. (2011). Strain-based topology optimization for crashworthiness using hybrid cellular automata. Int. J. Crashworth..

[36] Jia J., Da D., Loh C.-L., Zhao H., Yin S., Xu J. (2020). Multiscale topology optimization for non-uniform microstructures with hybrid cellular automata. Struct. Multidiscip. Optim..

[37] Tajs-Zielińska K., Bochenek B. (2023). Cellular Automata Approach to Topology Optimization of Graded Multi-Material Structures. Appl. Sci..

[38] Tajs-Zielińska K. (2024). Topology Optimization of Periodic Structures Subject to Self-Weight Loading Using a Heuristic Method. Materials.

[39] Zhang X., Wang D., Huang B., Wang S., Zhang Z., Li S., Xie C., Kong D. (2023). A dynamic-static coupling topology optimization method based on hybrid cellular automata. Structures.

[40] Lewiński T., Czarnecki S., Dzierżanowski G., Sokół T. (2013). Topologyoptimization in structuralmechanics. Bull. Pol. Acad. Sci..

[41] Sigmund O. (2001). A 99 line topology optimization code written in MATLAB. Struct. Multidiscip. Optim..

[42] Bendsoe M.P., Sigmund O. (2003). Topology Optimization: Theory, Methods and Applications.

[43] Bendsoe M.P. (1995). Optimization of Structural Topology, Shape, and Material.

[44] Bochenek B. (2023). An easy way for the generation of structural topologies under random loads using cellular automata. Acta Polytech. Hung..

[45] Roper S.W.K., Lee H., Huh M., Kim I.Y. (2021). Simultaneous isotropic and anisotropic multi-material topology optimization for conceptual-level design of aerospace components. Struct. Multidiscip. Optim..

[46] Trivers N.C., Carrick C.A., Kim I.Y. (2020). Design optimization of a business aircraft seat considering static and dynamic certification loading and manufacturability. Struct. Multidiscip. Optim..

[47] Liu K., Tovar A. (2014). An efficient 3D topology optimization code written in Matlab. Struct. Multidiscip. Optim..

